# Supplementary material for: A novel cellular structure in the retina of insectivorous birds
Source: Sci Rep. 2019 Oct 23;9:15230. doi: 10.1038/s41598-019-51774-w (PMC6811557; doi:10.1038/s41598-019-51774-w)
Supplement: Supplementary file 1 — Supplementary Materials [file 41598_2019_51774_MOESM1_ESM.pdf]

Supplementary Information for the article titled:

A novel cellular structure in the retina of insectivorous birds

Authors:

Luke P. Tyrrell[1]*, Leandro B.C. Teixeira[2], Richard R. Dubielzig[2], Diana Pita[3], Patrice Baumhardt[3], Bret A. Moore[4], Esteban Fernández-Juricic[3]

Affiliations:

[1]SUNY Plattsburgh, Department of Biological Sciences, 101 Broad Street, Plattsburgh, NY 12901

[2]University of Wisconsin-Madison, Department of Pathobiological Sciences, School of Veterinary Medicine, 2015 Linden Drive, Madison, WI 53706

[3]Purdue University, Department of Biological Sciences, 915 W State Street, West Lafayette, IN 47904

[4]University of California-Davis, William R. Pritchard Veterinary Teaching Hospital, School of Veterinary Medicine, 1 Garrod Drive, Davis, CA 95695

*Corresponding author: ltyrr002@plattsburgh.edu

Supplementary Table S1 | Microspectrophotometry of *Empidonax* flycatcher oil droplets

*No values are present for T-type oil droplets because they are transparent with no absorbance curve.

	T-type*	C-type	Y-type	R-type	P1-type	P2-type		MMOD-complex
						a	b	
Mean λ_{cut} (nm)	—	—	500 ± 1.83	560 ± 2.38	433 ± 2.48	449 ± 3.68	485 ± 1.26	543 ± 0.92
Mean λ_0 (nm)	—	—	512 ± 2.18	575 ± 1.86	451 ± 2.84	457 ± 2.83	495 ± 1.25	562 ± 0.91
Mean λ_{mid} (nm)	—	—	517 ± 2.31	581 ± 1.90	457 ± 3.11	459 ± 2.54	499 ± 1.26	569 ± 0.92
Mean b	—	—	0.088 ± 0.003	0.071 ± 0.005	0.065 ± 0.005	0.140 ± 0.014	0.105 ± 0.002	0.056 ± 0.001
Mean B_{mid}	—	—	0.030 ± 0.001	0.024 ± 0.002	0.022 ± 0.002	0.048 ± 0.005	0.036 ± 0.001	0.019 ± 0.000
λ_{cut} of mean absorbance spectrum (nm)	—	405	498	562	435	449	485	541
λ_0 of mean absorbance spectrum (nm)	—	412	511	575	447	456	495	559
λ_{mid} of mean absorbance spectrum (nm)	—	415	516	580	451	459	498	565
b of mean absorbance spectrum	—	0.150	0.080	0.082	0.090	0.139	0.114	0.060
B_{mid} of mean absorbance spectrum	—	0.052	0.028	0.028	0.031	0.048	0.039	0.021
n	1	1	16	8	13	4	—	23

Supplementary Table S2 | Statistics on oil droplet density ratios

Mean ± SE values can be found in Fig. 3a.

Comparison	Ratio	t-statistic	Degrees of freedom	p-value
Typical passerine versus *Empidonax*	C-type:T-type	0.33	8	0.752
	Y-type:T-type	0.87	8	0.411
	R-type:T-type	0.20	8	0.843
	P-type:T-type	0.16	8	0.876
	MMOD-complex:T-type	9.64	8	**< 0.001**
Empidonax periphery versus *Empidonax* center	C-type:T-type	0.67	3	0.725
	Y-type:T-type	3.61	3	0.982
	R-type:T-type	0.68	3	0.274
	P-type:T-type	1.21	3	0.059
	MMOD-complex:T-type	22.88	3	**< 0.001**

Supplementary Table S3. List of species used to construct a phylogeny of birds for which photoreceptor and oil droplet types are known. For statistical analyses, we only used species for which eye size and foraging data were also published; these species are denoted in the Used in Analysis column.

Family	Species	Source
Dromaiidae	*Dromaius novaehollandiae*	Hart et al. 2016
Struthionidae	*Struthio camelus*	Wright & Bowmaker 2001
Rheidae	*Rhea americana*	Wright & Bowmaker 2001
Anatidae	*Anas penelope*	Hart 2001
Anatidae	*Aythya affinis*	Hart 2001
Laridae	*Larus novaehollandiae*	Hart 2001
Laridae	*Anous minutus*	Hart 2001
Columbidae	*Stigmatopelia chinensis*	Hart 2001
Columbidae	*Columba livia*	Bowmaker et al. 1997
Alcedinidae	*Todiramphus sanctus*	Hart 2001
Cuculidae	*Eudynamys scolopaceus*	Hart 2001
Phasianidae	*Pavo cristatus*	Hart 2001
Phasianidae	*Gallus gallus*	Hart et al. 2006
Rallidae	*Gallunula tenebrosa*	Hart 2001
Phalacrocoracidae	*Phalacrocorax varius*	Hart 2001
Procellariidae	*Puffinus pacificus*	Hart 2001
Accipitridae	*Aquila audax*	Reymond 1985
Accipitridae	*Buteo buteo*	Mitkus et al. 2017
Accipitridae	*Pernis apivorus*	Mitkus et al. 2017
Accipitridae	*Milvus milvus*	Mitkus et al. 2017
Accipitridae	*Accipiter nisus*	Mitkus et al. 2017
Falconidae	*Falco berigora*	Reymond 1987
Falconidae	*Falco peregrinus*	Mitkus et al. 2017
Cacatuidae	*Cacatua roseicapilla*	Hart 2001
Psittaculidae	*Melopsittacus undulatus*	Knott et al. 2012
Psittaculidae	*Platycercus eximius*	Hart 2001
Psittaculidae	*Trichoglossus chlorolepidotus*	Hart 2001
Fringillidae	*Carduelis tristis*	Baumhardt et al. 2014
Turdidae	*Turdus migratorius*	Unpublished data
Turdidae	*Turdus merula*	Hart 2001
Turdidae	*Sialia sialis*	Unpublished data
Paridae	*Parus caeruleus*	Hart 2001
Meliphagidae	*Entomyzon cyanotis*	Hart 2001
Meliphagidae	*Manorina melanocephala*	Hart 2001
Icteridae	*Molothrus ater*	Fernandez-Juricic et al. 2013
Icteridae	*Agelaius phoeniceus*	Unpublished data
Icteridae	*Sturnella magna*	Tyrrell et al. 2013
Sturnidae	*Sturnus vulgaris*	Hart 2001
Passerellidae	*Zonotrichia albicollis*	This study
Passerellidae	*Spizella pusilla*	Unpublished data
Passeridae	*Passer domesticus*	This study
Ptilonorhynchidae	*Ptilonorhynchus violaceus*	Hart 2001
Cardinalidae	*Cardinalis cardinalis*	Unpublished data
Corvidae	*Cyanocitta cristata*	Unpublished data

Mimidae	*Dumetella carolinensis*	Unpublished data
Sittidae	*Sitta carolinensis*	Unpublished data
Tyrannidae	*Empidonax virescens*	This study
Tyrannidae	*Empidonax virescens*	This study

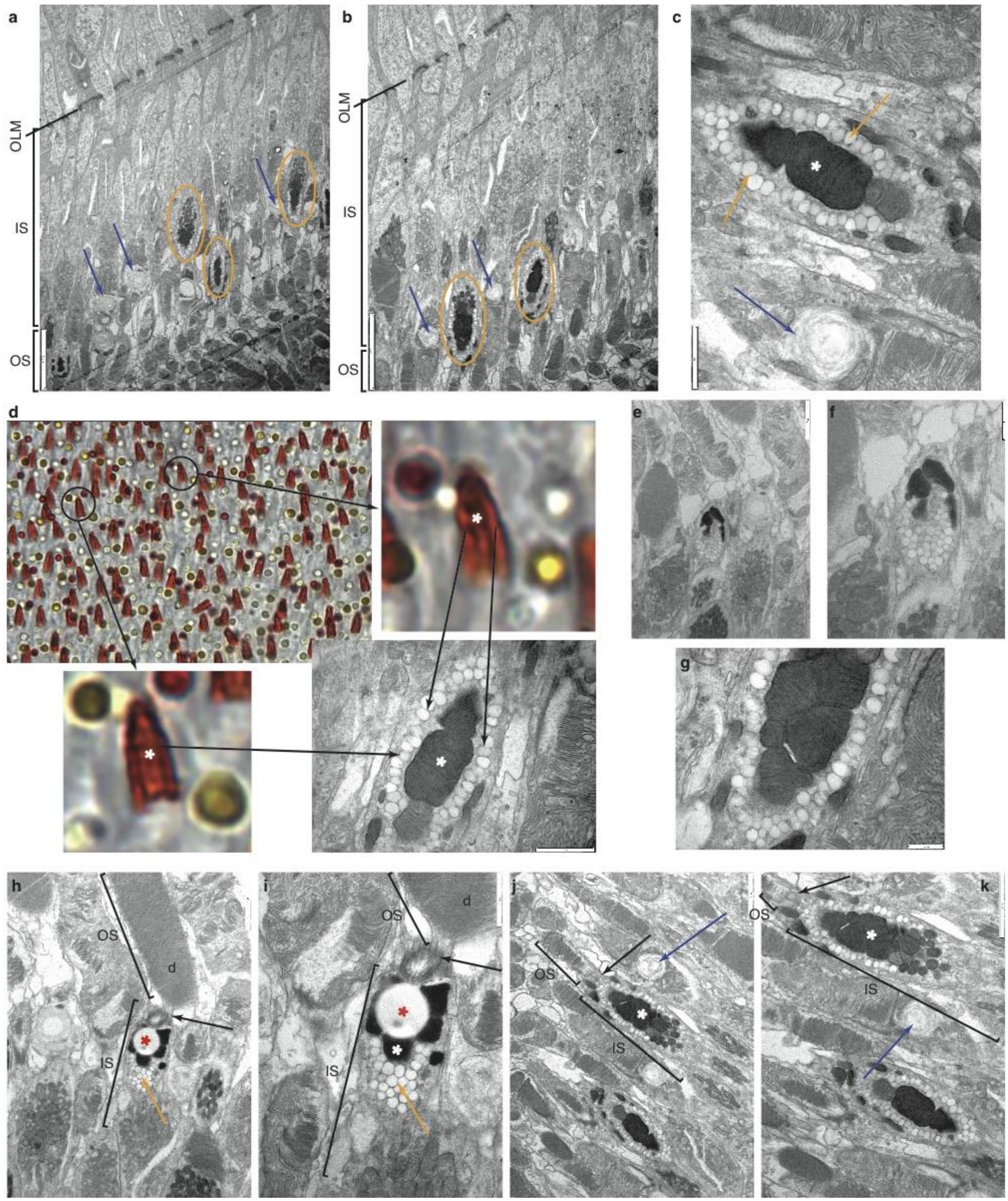

Supplementary Figure S1 | Images of MMOD-complex photoreceptors. (a-c, e-k)

Transmission electron microscopy of the MMOD-complex photoreceptors in *Empidonax*

flycatchers. (d) The shape, arrangement, and location of the MMOD-complex observed under transmission electron microscopy correlates with that of the wholemount under light microscopy. OLM refers to the outer limiting membrane, IS refers to the inner segments, OS refers to the outer segments, and *d* refers to photoreceptor discs. Orange ellipses encompass MMOD-complexes, orange arrows indicate the small oil droplets surrounding megamitochondria that confer the orange color on the wholemount, blue arrows indicate traditional oil droplets, white asterisks indicate electron-dense megamitochondria cores, and red asterisks indicate artifacts. Black arrows in (h-k) indicate connecting cilium between inner and outer segments. The MMOD-complexes shown were only present in the two samples form the center of an *Empidonax* retina. No similar structures were present in the peripheral *Empidonax* sample or any of the house sparrow samples.

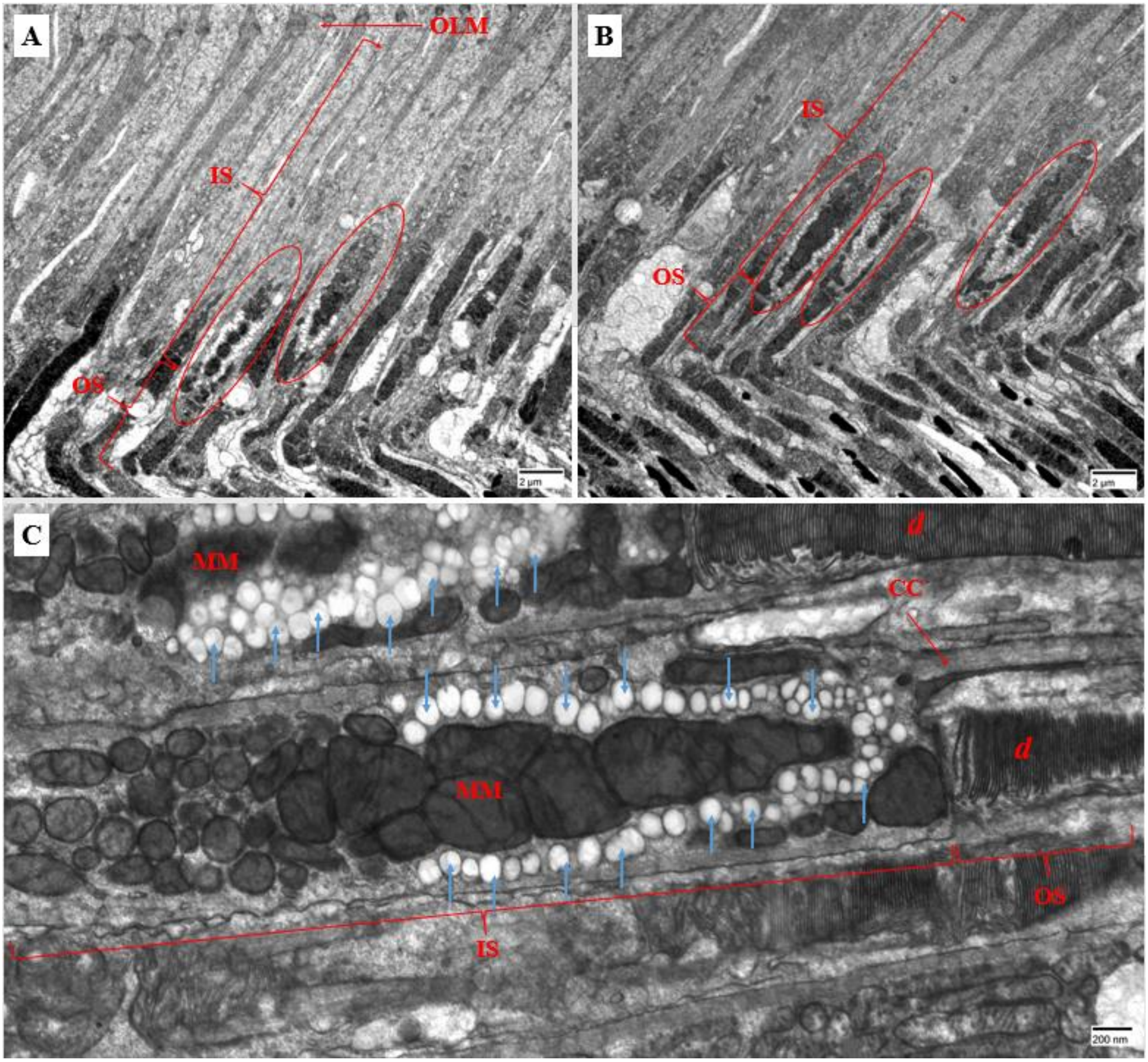

Supplementary Figure S2 | Images of MMOD-complex photoreceptors. (a-c). Transmission electron microscopy of the MMOD-complex photoreceptors in *Empidonax* flycatchers. **(a and b).** Multiple MMOD-complex (red ellipses) are observed in different areas of the central retina under transmission electron microscopy. OLM = outer limiting membrane, IS = inner segments of photoreceptors, OS= outer segments of the photoreceptors. **(c).** Higher magnification of an MMOD-complex photoreceptors highlighting the transition between the MMOD-complex in the photoreceptor inner segment (IS) and the photoreceptor discs (*d*) and connecting cilium (CC) in

the photoreceptor outer segment (OS). Blue arrows indicate the small oil droplets surrounding a megamitochondria (MM) that confer the orange color on the wholemount. No similar structures were present in the peripheral *Empidonax* sample or any of the house sparrow samples.

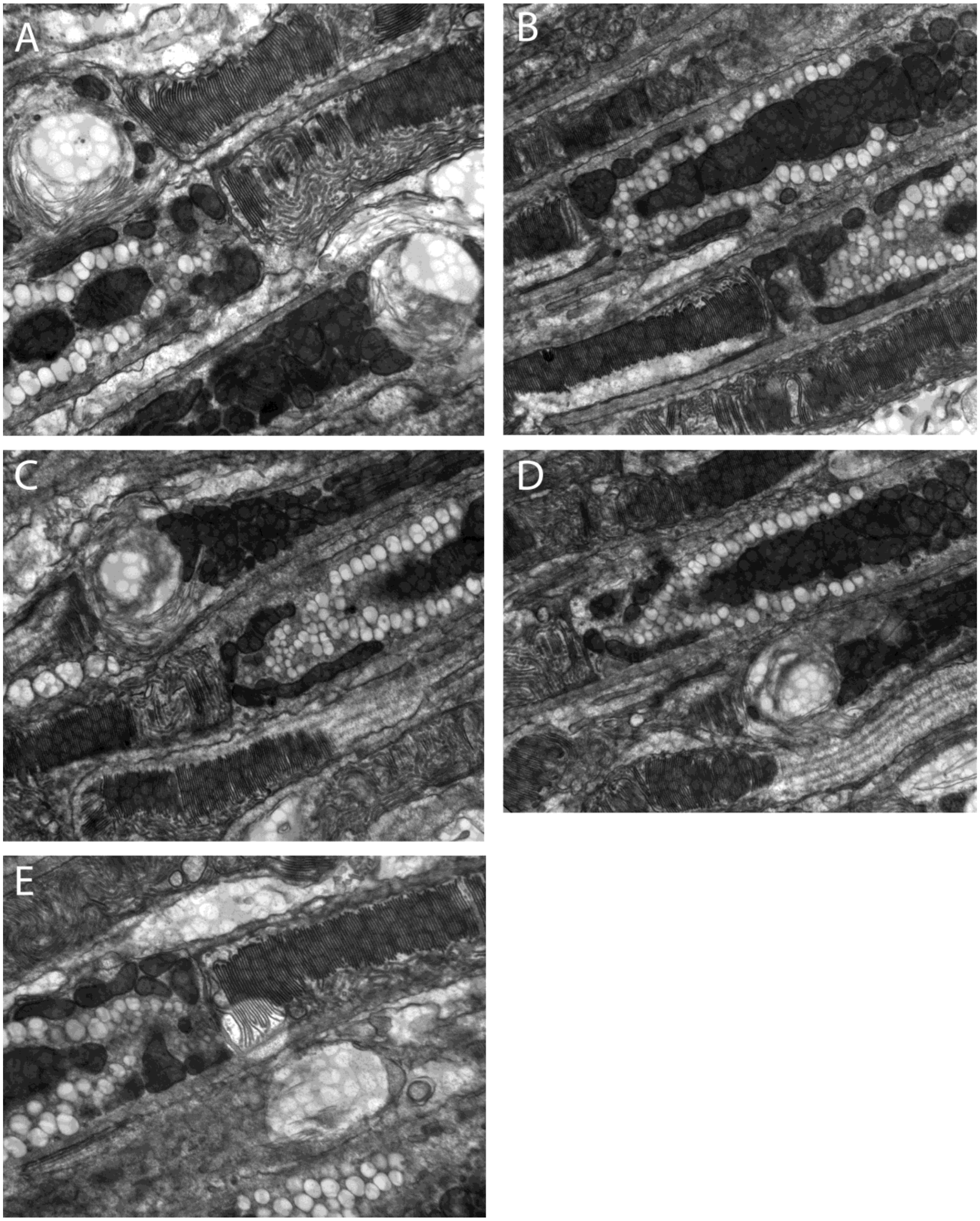

Supplementary Figure S3 | Additional Images of MMOD-complex photoreceptors. (a-e)

MMOD-complex photoreceptors imaged under transmission electron miscroscopy. Each image

depicts a different individual MMOD-complex photoreceptor that shows the MMOD-complex in the inner segment attached to a visual pigment-containing outer segment.

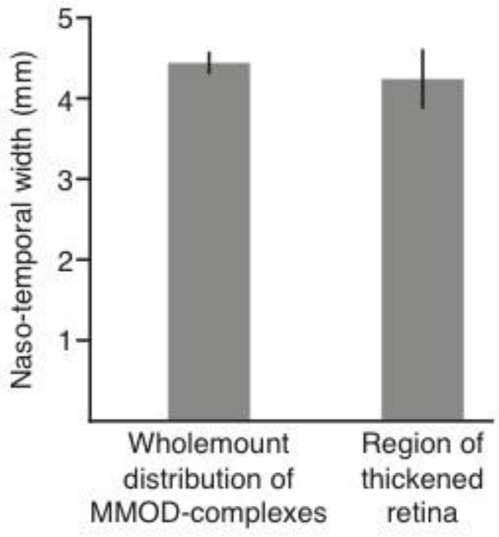

Supplementary Figure S4 | Retinal coverage of MMOD-complex photoreceptors. The distribution of MMOD-complex photoreceptors as measured on a retinal wholemount (image in Fig. 2j) corresponds to the thickened retinal region as measured on a cross section (image in Fig. 2k). This suggests that the MMOD-complex photoreceptors increase the demand for cells in other retinal layers, leading to a substantial thickening of the retinal tissue in regions where MMOD-complex photoreceptors are present. The MMOD-complex region covers the central 27.4 ± 1.7 mm^2 or 24.6 ± 0.9% of the *Empidonax* retina.
